# Systemic review of age brackets in pediatric emergency medicine literature and the development of a universal age classification for pediatric emergency patients - the Munich Age Classification System (MACS)

**DOI:** 10.1186/s12873-023-00851-5

**Published:** 2023-07-25

**Authors:** Alexander Althammer, Stephan Prückner, Geogr Christian Gehring, Victoria Lieftüchter, Heiko Trentzsch, Florian Hoffmann

**Affiliations:** 1 Institut für Notfallmedizin und Medizinmanagement (INM), Ludwig-Maximilians-University, Schillerstr. 53, 80336 Munich, Germany; 2 Department of Anesthesiology, Universitätsklinikum Augsburg, Stenglinstraße 2, 86156 Augsburg, Germany; 3 Pediatric Intensive Care and Emergency Medicine, Dr. von Hauner Children’s Hospital, Ludwig- Maximilians-University, Lindwurmstraße 4, 80337 Munich, Germany

**Keywords:** Age classification, Pediatric emergency, Age limits, Classify pediatric emergencies

## Abstract

Currently arbitrary, inconsistent and non-evidence-based age cutoffs are used in the literature to classify pediatric emergencies. None of these classifications have valid medical rationale. This leads to confusion and poor comparability of the different study results. To clarify this problem, this paper presents a systematic review of the commonly used age limits from 115 relevant articles. In the literature search 6226 articles were screened. To be included, the articles had to address the following three topics: “health services research in emergency medicine”, “pediatrics” and “age as a differentiator”. Physiologic and anatomic principles with reference to emergency medicine were used to solve the problem to create a medically based age classification for the first time.

The Munich Age Classification System (MACS) presented in this paper is thus consistent with previous literature and is based on medical evidence. In the future, MAC should lead to ensure that a uniform classification is used. This will allow a better comparability of study results and enable meta-analyses across studies.

## Introduction

Differentiation according to patient age is the most common method of distinguishing between pediatric and adult emergencies [1]. Up to now no uniform and internationally valid standard for the classification of pediatric patients on the basis of their age has been established [1, 2]. While age classification has been well studied for clinical trials [3, 2, 4], there is no detailed review for the field of epidemiological health services research. As a result, it is difficult to compare the results of individual epidemiologic papers to date and, consequently, overarching meta-analyses are possible only on a limited basis. It is therefore imperative to agree internationally on an age classification that is as uniform as possible for future work.

The goal of this review is therefore the identification of different age groups in pediatric emergency care. We first reviewed the classifications found in the literature and identified differences. Then, based on physiological and anatomical conditions, we created our proposal for a unified classification from the previously reviewed categories. Thus, the age classification presented in this text is intended to serve as an internationally uniform reference for further studies in the future.

## Methods

We conducted a systematic literature review using the PRISMA method [5]. The research question was addressed using the *PICO* scheme as follows [6]:

### **P**roblem

inconsistent age classification of pediatric emergencies to date.

**I**ntervention: relevant articles were first identified based on an extensive literature search and the age classifications used were examined in more detail. The articles had to address the three aspects of “age as a differentiator,“ “health services research in emergency medicine,“ and “pediatrics” to be included in the literature selection process. For this purpose, various individual terms and so-called “MESH terms” were combined into different queries of the *Pubmed (MEDLINE)* database. “MESH terms” are terms defined by the database to better categorize and classify individual articles. Table 1 lists all queries that were used for the literature search and indicates how many hits were found and how many articles were included in the final evaluation.

**Table 1:** Queries used in the literature search}

Query	Date	Hits	Used
Adolescent[MeSH Terms] AND Emergency Service, Hospital/statistics and numerical data[MAJR]	April 3, 2020	4472	51
Age Factors[Mesh]) AND pediatric emergency	April 2, 2020	1321	36
(Adolescent/physiology[MeSH Terms]) AND Age Factors[MeSH Terms]	April 7, 2020	72	9
Difference*[Title/Abstract]) AND Pedia- tric*[Title/Abstract]) AND Adult* [Title/Abstract]) AND Emergency*[Title/Abstract]	April 7, 2020	352	15
Triage[MeSH Terms]) AND Child[MeSH Terms]) AND Intensive Care Units, Pediatric“[MeSH Terms]) AND Emergency Treatment[MeSH Terms]	April 7, 2020	9	4

**Fig. 1:**
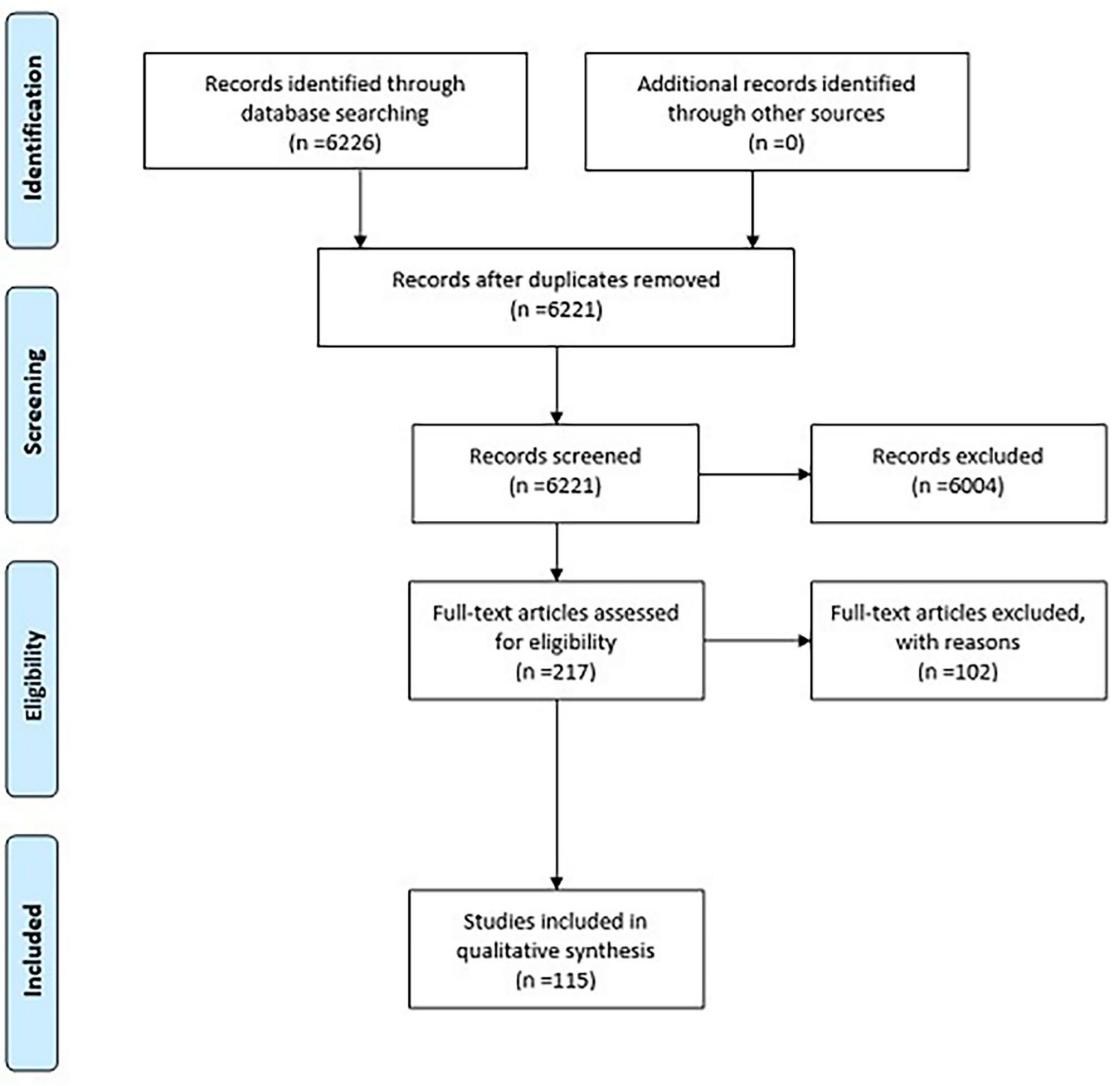
PRISMA flowchart for the literature search

As Fig. 1 shows, the initial query produced 6,226 hits. After duplicates were removed, the texts were checked for relevance and topicality based on the publication date, the title and the abstract. Accordingly, only 217 titles were evaluated as suitable for further consideration. All 217 titles dealt with a topic that contributes to answering the initial question and were published after 1980. Only texts that defined clear age limits were used for further analysis. Thus, from an initial 6226 articles found, 115 could be filtered for final analysis.

**C**omparison: results from the literature search will be compared with two particularly relevant already existing proposals for age classification. The proposed age groupings of the *National Association of Statutory Health Insurance Physicians for Germany* and the *Eunice Kennedy Shriver***N**ational **I**nstitute of **C**hild **H**ealth and **H**uman **D**evelopment NICHD for the English-speaking world were used as a reference [3],[7].

**O**utcome: development of the final age classification. In order to create a generally valid classification, selected developmental steps of each child and the associated physiological and anatomical changes in childhood are examined. The aim is to use suitable examples to show fundamental differences in the emergency medical care of children and adults as a function of age. These differences will be used to establish a consistent and well-reasoned age classification of pediatric emergencies based on the results of the literature review.

## Results

### Intervention: analysis of the identified articles

The final 115 articles are evaluated and analyzed below. To get an overview of age limits already in use within pediatric emergency care, the age limits from the 115 articles were aggregated and examined according to their frequencies.

**Fig. 2:**
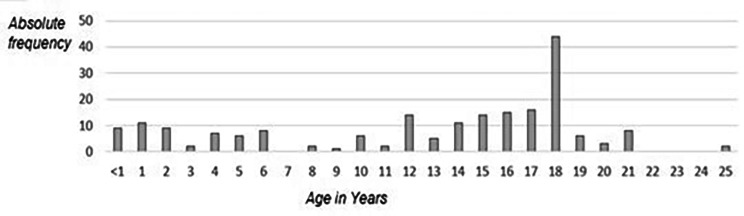
Age distribution within the literature search

Figure 2 reveals a separation into five groups:

- Group 1: ≤ 1–2 Years.
- Group 2: 3–6 Years.
- Group 3: 7–12 Years.
- Group 4: 13–17 Years.
- Group 5: ≥ 18 Years.

It should be noted that the sum of the individual characteristics exceeds the article number of 115, since in several articles not just one age was considered as a limit, but there were staggered intervals with several subgroups. To illustrate this fact, articles that consider subgroups were analyzed separately.

**Fig. 3:**
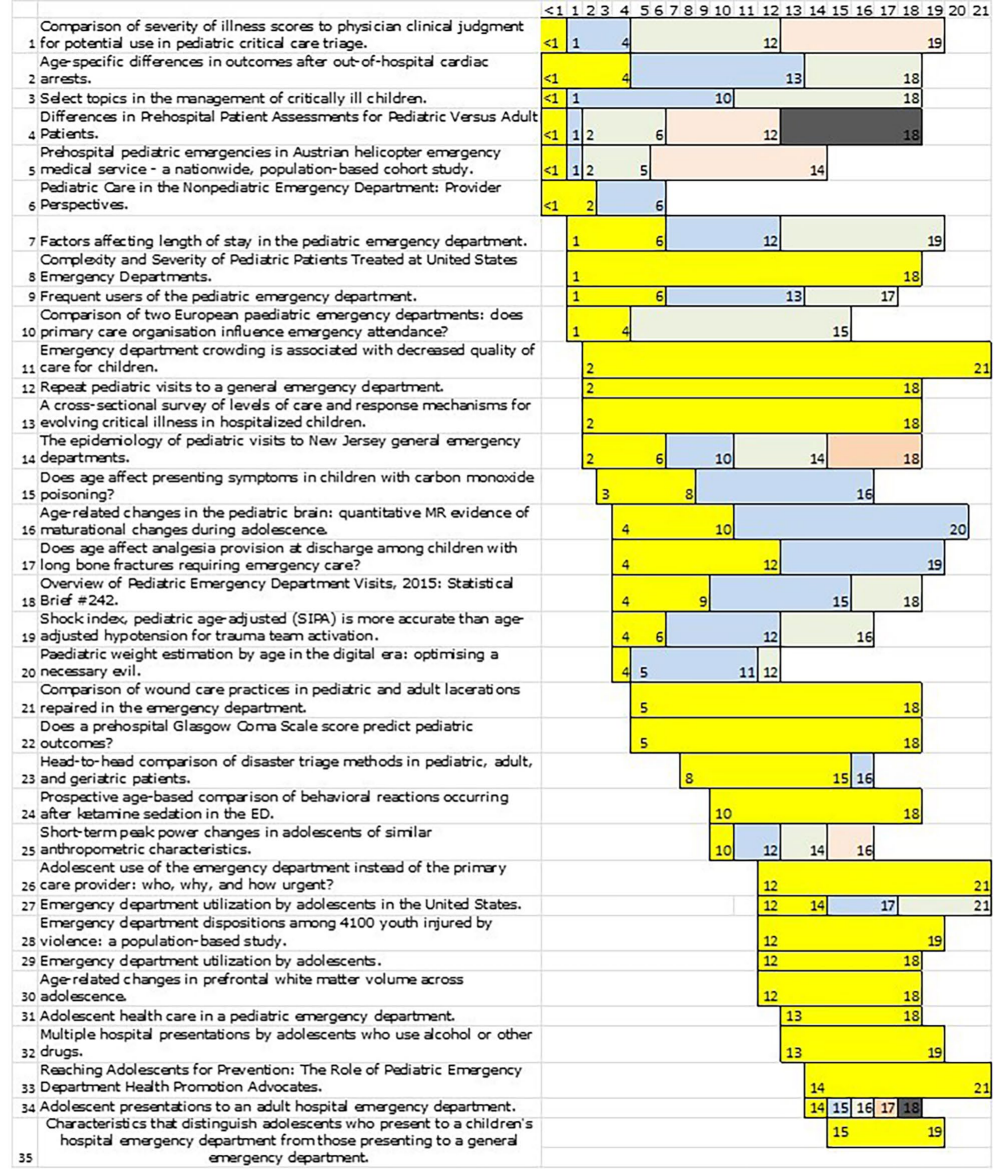
Subgroups from the literature

Figure 3 illustrates the distribution of these subgroups graphically. Most of the articles do not form subgroups, but commit themselves to a fixed age limits for differentiating between childhood and adulthood. Only 35 of the 115 articles examined considered further subdivisions in their work. Of these 35 articles, 15 in turn use only one subdivision using two age groups. Figure 3 primarily shows that no uniform approach can be identified with regard to age limits. It can be seen that patient ages of < 1, 2, 6, 12 and 18 years were used particularly often for classification.

Comparison: Comparison between the result of the literature review and national recommendations.

The following classification is one of the most common used in Germany [7].

- Newborn: up to the completed 28th day of life.
- Infant: 29 days – 12 months.
- Toddler: 2–3 years.
- Child: 4–12 years.
- Adolescent: 13–18 years.
- Adult: from the beginning of the 19th year,

while the classification of the Eunice Kennedy Shriver National Institute of Child Health and Human Development (NICHD) frequently used in the U.S. and Australia: [3]

- Infancy: Birth – 12 months.
- Toddler: 13 months – 2 years.
- Early childhood: 3–5 years.
- Middle childhood: 6–11 years.
- Early adolescence: 12–18 years.
- Late adolescence: 19–21 years.

While the NICHD’s recommendation differentiates in some respects in more detail than the common used classification in Germany, significant similarities nevertheless emerge: First, the termination of infant status at 12 months is consistent. Similarly, the age limit of 18 years is present in both cases. The difference in middle childhood is interesting. The recommendation from the U.S. provides for separation at 6 years. The National Health Insurance Association includes this age group undifferentiated with the interval of 4–12. It is also not clear from Fig. 2 whether differentiation is more common in the existing literature for 6- to 11-year-olds or for 4- to 12-year-olds.

### Outcome: development of the final age classification

This part of our work addresses particularly relevant aspects in the treatment of pediatric emergency patients. Together with the basic physiological and anatomical characteristics presented below, the proposed new age classification was established. For the following reasons, we focus on the following three topics:

- The clinical picture of sepsis is found at the top of the most common causes of death in children worldwide [8]. Fever as a leading symptom of sepsis in childhood serves as a motivation to go into more detail on the development of the immune system [9].
- Respiratory emergencies are among the most common emergency situations, especially in children [10].
- In 2014, 56,800 deaths related to traumatic brain injury were recorded in the USA, 2,529 of which involved children [11]. Epidemiological studies for Germany showed that the incidence of traumatic brain injuries is above average, especially in patients under 16 years of age [12]. Furthermore, it was found that patients who had not yet completed their first year of life had a twice as high incidence of traumatic brain injury compared to the general population [12].

Table 2 briefly summarizes the most important age limits from the selected examples. It can be seen that newborns represent a group of their own. Children up to 2 years of age also show some distinctive features. At the age of 5, the next clear developmental step can be seen, before puberty begins at around 11. It is clear that in a generally valid age classification a demarcation within the age of 4–12 years is indispensable. By the age of 18, most vital signs and anatomical conditions are at the level of an average adult.

**Table 2:** Summary of the relevant age limits

Special features in pediatric emergency		
Sepsis, Temperature & Immune System	Greater body surface & thinner skin, heat generation by brown adipose tissue [13]	Newborn
	Special diagnostic scheme for elevated temperature [13]	Newborn
	Postoperative muscle tremor for heat generation [13]	Starting from 6 years
	Monocytes restricted to few cytokines [14]	Newborn
	Immune system more in an anti-inflammatory mode [14]	Up to 3 years
	Differentiation of B lymphocytes [15]	Up to 5 years
	Differentiation of the innate immune system [14]	Starting from 5 years of age - approx. 13 years of age
Respiration	Greatest formation of new alveoli [16]	Up to the age of 2
	Completion of alveolar formation[16]	Starting from 12 years
Traumatic brain injury	Unclear oncogenic effect unsuitable for initial assessment [17]	Up to 2 years
	Glasgow coma scale unsuitable for initial assessment [18]	Up to 3 years
	Immature cranial calvaria, higher water content, heavy head & weak musculature [19]	Up to 5 years
	Often different accident mechanism - often involved as pedestrian in the accident [20]	Up to 6 years
	“Kennard-Principle” Better outcome with traumatic brain injury [21]	Up to 12 years

## Discussion

Our literature review shows that currently mostly an arbitrary and often insufficiently justified classification of the studied population is made on the basis of age.

The aim of this work was therefore to establish an internationally applicable age classification for pediatric emergencies. Although Clark et al. [3] primarily referred to the exact terminology of the child within medicine and Williams et al. dealt with the age classification for clinical studies [2], the aim of this work was to clearly review the classifications used so far in the literature for the first time. The basic physiological and anatomical differences that are instrumental in differentiating patients, particularly in emergency medicine, were used to create and justify a reasonable classification.

The following classification of the different pediatric ages, shown in Table 3, is proposed as the Munich Age Classification System:

**Table 3:** Munich Age Classification System (MACS)

Term	Age	Weight 50th percentile in kg [22]
**Neonate**	Up to the 27th day of life	3,3 – ≤ 4,5
**Infant**	30 days – 12 months	4,5 – ≤ 9,9
**Toddler**	13 months – 2 years	9,9 – ≤ 15
**Early childhood**	3 years – 5 years	15 – ≤ 21
**Late childhood**	6 years – 11 years	21 – ≤ 41
**Adolescent**	12 years – 17 years	41 – ≤ 64
**Adult**	Older than 17 years	> 64

Particular attention should be drawn to the differentiation between early and late childhood. This subdivision is not found in the recommendation of the Association of Statutory Health Insurance Physicians. However, as in Table 2 could be seen we have been able to determine treatment-relevant age-dependent differences exactly for this period. The immune system reaches a new physiological developmental stage (differentiation of B-lymphocytes) at around 5 years of age. This leads to the immune system being able to work more specifically and no longer having to respond to known pathogens with a generalized immune response. The anatomy of the skull changes in a way that results in relevant differences in treatment in case of trauma, and the mechanisms of accidents also differ from each other at around 6 years of age.

Furthermore, the weight distribution of MACS reveals significant differences in weight within the two age categories. As medication dosages are weight dependent, this provides further justification for stratification within the age range of 3–11 years.

A differentiation is therefore strongly recommended at this point. Our research presents a unified classification based on the existing literature as well as selected anatomical and physiological peculiarities. Existing clinical recommendations are often described inconsistently and use different distinguishing features. The relevance of the MACS to individual clinical procedures (such as resuscitation, intubation, analgesia, ventilation, wound care, clinical imaging) represents a research prospect for further studies. It is recommended to decide the assignment of the patient to the appropriate category either according to the age of the MACS or according to the corresponding weight as shown in Table 3.

The greatest limitation of this work is the selective choice of topics with respect to physiological and anatomical differences. The focus on emergency medicine is evident in the selection of topics. Therefore, other parameters such as the onset of sexual maturity, the change in metabolism or the hormonal transition of the body were not addressed. Furthermore, it should be noted that the aggregation and categorization of patients based on age inevitably leads to inaccuracies and not all details can be represented. In particular, individual characteristics or certain details of specific research questions cannot always be mapped with this. Willimans et al. showed, at least for clinical studies, how a generally applicable age classification could best be adapted to individual research questions [2]. Legal and administrative regulations, such as age of compulsory education or attainment of full legal capacity, also vary both nationally and internationally. It is therefore not realistic to map all relevant factors in a universally valid classification. However, it is much more important that a uniform classification is used despite of these limitations - even if this does not describe all details. Only in this way is it possible to evaluate research results as efficiently as possible and without diminishing their significance, even across international boundaries. Consequently, it is not important that the classification used represents reality in its entirety, but rather that there is international agreement on a uniform standard. The age classification of this work can thus contribute to counteracting the current practice of strongly varying and often arbitrary classifications of patients.

## Data Availability

All data generated or analysed during this study are included in this published article.
