# Correction: Systemic review of age brackets in pediatric emergency medicine literature and the development of a universal age classification for pediatric emergency patients - the Munich Age Classification System (MACS)

**DOI:** 10.1186/s12873-024-01064-0

**Published:** 2024-08-09

**Authors:** Alexander Althammer, Stephan Prückner, Geogr Christian Gehring, Victoria Lieftüchter, Heiko Trentzsch, Florian Hoffmann

**Affiliations:** 1 Institut für Notfallmedizin und Medizinmanagement (INM), Ludwig-Maximilians-University, Schillerstr. 53, 80336 Munich, Germany; 2 Department of Anesthesiology, Universitätsklinikum Augsburg, Stenglinstraße 2, 86156 Augsburg, Germany; 3 Pediatric Intensive Care and Emergency Medicine, Dr. von Hauner Children’s Hospital, Ludwig- Maximilians-University, Lindwurmstraße 4, 80337 Munich, Germany

**BMC Emergency Medicine (2023) 23:77**

10.1186/s12873-023-00851-5

The original article contains errors in the proposed age classification relating to missing days between Neonates and Infants, unclear distinction between age brackets, and unclear end of adult age bracket.

An updated version of the mMACS can be viewed ahead in this Correction article.

Furthermore, the amendments noted above were suggested by Daniel Pfeiffer of the Dr. v. Hauner Children’s Hospital, Munich, Germany who is acknowledged via this Correction article.

**Table 1:** Queries used in the literature search

Term	Age
**Neonate**	≤ 28 days
**Infant**	≥ 29 days – < 1 year
**Toddler**	≥ 1 year - < 3 years
**Early childhood**	≥ 3 years – < 6 years
**Late childhood**	≥ 6 years – < 12 years
**Adolescent**	≥ 12 years – < 18 years
**Adult**	≥ 18 years

